# Challenges and perspectives for internal radiation exposure examinations using the whole-body counting system following the Fukushima Daiichi nuclear power plant accident

**DOI:** 10.3389/fpubh.2026.1904248

**Published:** 2026-07-27

**Authors:** Takashi Ohba, Isamu Amir, Kazuki Yoshida, Haruka Furuiwa, Michio Murakami, Masaharu Tsubokura

**Affiliations:** 1 Department of Radiological Sciences, Fukushima Medical University School of Health Sciences, Fukushima, Japan; 2 Takemi Program in International Health, Harvard T. H. Chan School of Public Health, Boston, MA, United States; 3 Department of Radiation Health Management, Fukushima Medical University School of Medicine, Fukushima, Japan; 4 College of Nursing, Iwate University of Health and Medical Sciences, Morioka, Japan; 5 Department of Radiation Disaster Medicine, Fukushima Medical University School of Medicine, Fukushima, Japan; 6 Center for Infectious Disease Education and Research, The University of Osaka, Suita, Japan

**Keywords:** Fukushima nuclear accident, internal radiation exposure, preparedness and response, radio-cesium, residents, risk communication, stakeholder, whole body counting system

## Abstract

Radiation protection is a fundamental component of public health crisis management. Following the Fukushima Daiichi nuclear power plant (FDNPP) accident, relevant authorities have conducted internal radiation exposure examinations (IREEs) for residents for more than 15 years as part of ongoing public health response efforts. This report identifies three major challenges in evaluating the continued implementation of IREEs: (i) determining why IREEs have been sustained despite the extremely low proportion of residents with internal radiation exposure >1 mSv; (ii) examining how large-scale IREEs using whole-body counting systems have been implemented in practice; and (iii) evaluating how stakeholder engagement in radiation risk communication has contributed to both safety and security in Fukushima. These challenges and perspectives encompass numerous “Lessons learned from Fukushima.” Three key perspectives were identified: (i) improving understanding of residents' perceptions of IREEs and identifying an appropriate balance between service provision and community demand to ensure equitable access; (ii) promoting the sustainable implementation of IREEs, strengthening preparedness for future nuclear disasters, and preserving Fukushima's long-term experience; and (iii) clarifying the current status and effectiveness of radiation risk communication associated with IREEs. To address these issues, a new initiative, the “Establishing Resident-Centered Implementation Policies for Internal Radiation Exposure Examinations” (RECIPEx) project, was launched in Japan from April 2025 to March 2028. The RECIPEx project is expected to provide a practical framework for integrating protection against internal radiation exposure into disaster preparedness strategies, thereby strengthening health management systems through the application of “Lessons learned from Fukushima.”

## Introduction

In the event of a major nuclear accident involving the release of radioactive materials beyond a nuclear facility, radiation protection measures must be implemented to prevent deterministic effects (tissue reactions) and reduce the risk of stochastic effects (1). The World Health Organization (WHO) (2) recognizes radiation protection as a public health measure within health crisis management frameworks. Protective actions for residents following a major nuclear accident include evacuation, temporary relocation, and sheltering (3). Such incidents may result in both external and internal exposure. To minimize internal exposure, measures are implemented to prevent the inhalation and ingestion of radioactive materials.

Following the Fukushima Daiichi nuclear power plant (FDNPP) accident, the principal radionuclides released were ^131^I (physical half-life: 8.03 days), ^134^Cs (physical half-life: 2.07 years), and ^137^Cs (physical half-life: 30.1 years) (4). Exposure to ^131^I raised concerns regarding increased thyroid doses in children, based on evidence from the Chornobyl NPP accident linking childhood thyroid exposure to elevated thyroid cancer risk (5). Additionally, radio-cesium isotopes, including ^134^Cs and ^137^Cs, were expected to contribute to prolonged internal exposure because of their relatively long physical half-lives (4). Since the FDNPP accident, food products exceeding regulatory limits for radio-cesium have primarily included wild vegetables, mushrooms, and meat from wild animals inhabiting forested areas within approximately 50 km of the FDNPP (6). Consequently, even 15 years after the FDNPP accident, some Fukushima residents continue to exercise caution regarding the consumption of specific foods and water as part of radiation protection measures against internal exposure.

Individual monitoring can provide clear evidence of radiation exposure levels and support health management following a nuclear disaster (1). One approach for assessing internal radiation exposure is the external measurement procedure using a whole-body counting (WBC) system (1, 4). WBC systems detect gamma rays emitted from radioactive materials such as ^131^I, ^134^Cs, and ^137^Cs within the human body. Within the first year after the FDNPP accident, some residents underwent WBC measurements for ^131^I and radio-cesium (7). Subsequently, monitoring efforts focused exclusively on radio-cesium, and residents who requested examinations continued to undergo assessments of internal exposure from ingestion for 15 years after the accident (8, 9).

Among residents who underwent internal radiation exposure examinations (IREEs) using WBC systems, only approximately 0.01% recorded internal radiation exposure levels >1 mSv following the FDNPP accident (10). The main cause of internal radiation exposure in these affected populations was consumption of wild vegetables and mushrooms growing in forested areas contaminated with radioactive cesium released during the FDNPP accident, as well as meat from wild animals living in those forests (10). On the other hand, this low proportion is largely attributable to regulatory controls on radioactive material concentrations, including radio-cesium in food products. This observation gives rise to the first challenge: why have IREEs continued for more than 15 years after the FDNPP accident despite the low frequency of exposures >1 mSv? Addressing this question requires examination of the rationale for maintaining IREEs from the perspectives of both the authorities responsible for their implementation and the residents who seek these examinations. A critical assessment of the current status of IREEs is also necessary.

Additional issues warrant further investigation. The second challenge concerns the implementation of IREEs. Following the FDNPP accident, residents were able to undergo IREEs free of charge through the local authorities in which they were registered, provided they wished to be examined (8, 9, 11). Organizations responsible for implementing IREEs, including Fukushima Prefecture, rapidly established systems capable of conducting large-scale examinations within approximately 2–3 years after the accident, with the goal of assessing as many residents as possible (12). Although different institutions used WBC systems from various manufacturers and models, no standardized procedures were established across facilities. Nevertheless, large-scale IREEs in Fukushima were conducted efficiently and accurately. To inform preparedness for future nuclear disasters, it is necessary to clarify the factors that enabled the successful implementation of these large-scale examinations.

The third challenge concerns stakeholder involvement. Stakeholders, defined as individuals, groups, or organizations with an interest in or concern about an issue, including experts and healthcare professionals (1), who have been explaining IREE results in Fukushima (13). Beyond reducing internal radiation exposure and thereby enhancing safety, IREEs also contributed to residents' sense of security through interactions with these stakeholders (11, 13). Understanding how IREEs promoted both safety and security among residents is therefore an important area of investigation.

This report identifies three key challenges: (i) the rationale for the continued implementation of IREEs; (ii) the operational practices underlying WBC-based examinations; and (iii) stakeholder involvement in the IREEs. By examining these issues more than 15 years after the FDNPP accident, the report presents perspectives for a distinctive analysis and contributes to the development of guidelines for authorities responsible for IREEs in future nuclear disaster responses.

## Challenges identified in IREEs for residents in Fukushima and perspectives for further investigation

Three key challenges associated with IREEs for residents utilizing WBC systems were identified following the FDNPP accident. Drawing on long-term experience with IREE in Fukushima, three interrelated challenges have become increasingly evident as the program has transitioned from large-scale post-accident examinations to a long-term, low-volume, request-based service. First, the marked decline in examination frequency has resulted in an imbalance between available examination capacity and current resident demand. Second, the reduction in examination volume has increased the risk of losing the practical expertise and operational knowledge accumulated by WBC operators. Third, although IREEs have served not only as tools for dosimetric assessment but also as opportunities for face-to-face risk communication, these communication practices have not been adequately documented or systematized. These three challenges with IREEs for residents utilizing WBC systems involve the transfer of knowledge strategy (14). The transfer of knowledge strategy refers to the systematic documentation, preservation, and sharing of practical knowledge and operational experience gained from IREEs conducted after the FDNPP accident in this report. This strategy will pass on knowledge regarding IREEs among affected populations in Fukushima, operational procedures of the WBC system, and the methodologies for radiation risk communication with stakeholders. Furthermore, this strategy will strengthen preparedness for future nuclear disasters and help disseminate valuable lessons learned from long-term experience in Fukushima to the international community.

These observations highlight three priority areas for future investigation: achieving an appropriate balance between examination supply and resident demand, preserving the operational know-how, and clarifying the contribution of stakeholder engagement.

### Challenge 1: discrepancy between supply and demand caused by the quantity of IREEs and the current state of WBC system installations

The IREEs for residents in Fukushima Prefecture were initiated in JFY (Japanese fiscal year) 2011 and have continued since then. The IREEs are administered by the Ministry of the Environment (MOE) and Fukushima Prefecture and are conducted not only by Fukushima Prefecture but also by Non-Profit Organizations (NPO)'s and medical institutions. However, an imbalance between supply and demand has emerged. According to reports from Fukushima Prefecture and the MOE, the annual number of IREEs exceeded 90,000 in JFY 2012 but declined to approximately 850 by JFY 2025 (15, 16). Following the FDNPP accident, approximately 50 WBC systems were installed throughout Fukushima Prefecture to support large-scale examinations (12). However, as IREE frequency has decreased, many WBC systems have become inactive. Consequently, given the number of WBC systems and the current volume of IREEs, examination capacity exceeds present demand. Under these circumstances, a discrepancy in perceptions regarding IREEs may exist between the authorities providing the examinations and the residents receiving them. Therefore, it is necessary to clarify the balance between supply and demand within the current IREE system.

In addition, WBC systems that are no longer operational could be repurposed for other applications, such as nuclear disaster training. Another potential application is the measurement of a whole-body skeletal muscle mass using radioactive potassium (17). During the recovery phase following a nuclear disaster, the agencies responsible for installing WBC systems should actively collect and demonstrate ways to utilize systems that are no longer operational.

#### Perspective 1: clarifying the balance between supply and demand for IREEs

The IREEs for residents are still in operation, albeit on a reduced scale, as described in Challenge 1 (15, 16). If IREEs were discontinued entirely in Fukushima, residents wishing to undergo such examinations might experience uncertainty. Therefore, maintaining access to IREEs while ensuring an appropriate balance between supply and demand is essential. Determining this balance requires clarification of residents' perceptions of and needs for IREEs through qualitative research, while also estimating future demand using chronological records of IREE implementation in each region of Fukushima Prefecture.

To achieve this objective, qualitative research should include interviews with (i) individuals who have continued to undergo IREEs since the accident, (ii) residents who underwent examinations after the accident but no longer participate, and (iii) individuals who have never undergone an examination. Additionally, large-scale questionnaire surveys are needed to assess the current status of past IREE records and the extent to which examination results are understood. Furthermore, future demand projections should be developed based on the number of IREEs conducted in Fukushima Prefecture to date. These investigations are expected to clarify residents' perceptions of IREEs and help identify an appropriate balance between supply and demand, ensuring that residents from diverse backgrounds can access such examinations.

Ultimately, if the WBC system consistently yields results below the detection limit for the same resident and there is negligible risk of internal exposure in their living area, this may justify the conclusion that IREEs will no longer be necessary in the future. Therefore, this information should be used to help residents understand the rationale for discontinuing this examination.

### Challenge 2: absence of operational procedures for large-scale IREEs in Fukushima

The accuracy of internal radiation exposure dose assessments using WBC systems depends on appropriate operational procedures and the expertise of staff at each facility. Various WBC system types have been installed in Fukushima Prefecture, including standing-type, sitting-type, bed-type, and BABYSCAN^®^ (a pediatric bed-type) (8, 9, 11, 12). To ensure efficient operation, the minimum detectable activity (MDA) for radioactive cesium is recommended to be < 300 Bq per body, with measurement times ranging from 2 to 5 minutes depending on the WBC systems (9, 12, 18). Thus, the measurement method for assessing internal radiation exposure doses using WBC systems was set by the detection limit for radioactive cesium. Some standing-type and sitting-type WBC systems were installed in mobile units, such as buses (12). Mobile units installed with WBC systems could be transported to areas with many affected populations. However, in areas with high environmental background radiation due to radioactive cesium released by the FDNPP accident, shielding provided by mobile WBC units was sometimes insufficient for IREE measurements, resulting in readings in which the MDA was elevated (19). Furthermore, the IREEs in Fukushima covered not only adults but also children among the affected population. For children's IREEs, not only were measurements of radioactive cesium with an MDA of < 50 Bq performed using BABYSCAN^®^, but measurements were also taken using a standing-type WBC system, with children under 140 cm in height standing on a 30 cm height step stool (11, 12). This method was designed to overestimate the amount of radioactive cesium by assuming that children were over 140 cm in height, as the internal radiation exposure of people under 140 cm in height tends to be underestimated in a standing-type WBC system (12). No age-based calibration for children was performed for children's IREEs in Fukushima as overestimation results in conservative estimates of internal radiation exposure.

In addition to methods for determining the detection limit of radioactive cesium, various approaches have been used to accurately assess internal radiation exposure doses with WBC systems according to the operational procedures of individual facilities. For example, one report suggested that internal radiation exposure doses can be measured accurately by eliminating surface contamination (18). Through these practical techniques, large-scale IREEs were successfully conducted in Fukushima Prefecture. However, as IREE scope has declined, the number of staff involved has also decreased. Consequently, practical knowledge by staff conducting IREEs are gradually being lost. These operational insights represent invaluable information for preparedness and response to future nuclear disasters. Therefore, sharing this knowledge is essential to support the accurate measurement of internal radiation exposure doses using WBC systems. Ultimately, all procedures within the WBC system will be documented in a well-considered manner, which will lead to the implementation of a quality management system. Furthermore, facilities with a WBC system should be encouraged to verify the validity of their measurement methods through interlaboratory comparisons. Additionally, under these conceptions, the transfer of knowledge strategy will refer to the systematic documentation, preservation, and sharing of practical knowledge and operational experience gained from IREEs.

#### Perspective 2: passing on the knowledge and experience accumulated through the IREEs

The knowledge and experience gained through IREEs, which embody “Lessons learned from Fukushima,” should be passed on to future generations as part of preparedness for future nuclear disasters. Currently, facilities conducting IREEs maintain their own examination procedures and reports, some of which include facility-specific operational practices (12, 16). By compiling this information, practical knowledge specific to IREE implementation can be incorporated into future manuals for IREEs following nuclear disasters. The systematic documentation and preservation of operational know-how accumulated through IREEs would support the sustainable implementation of IREEs and future nuclear disaster preparedness. Additionally, it would enable Fukushima's long-term experience to be shared internationally as a valuable source of knowledge.

### Challenge 3: lack of systematic documentation of radiation risk communication practices related to IREEs

The IREEs conducted in Fukushima were intended to ensure safety based on examination results and provide security for residents (11, 13). For example, residents who consumed self-harvested foods, including vegetables or wild plants collected from mountainous areas, could undergo IREEs to assess their radiation exposure doses and confirm their safety (20). Furthermore, these examinations contributed to the dissemination of radiation-related knowledge and enhanced residents' awareness of radiation risks (13). Specifically, IREEs functioned as a form of radiation risk communication between stakeholders responsible for explaining radiation exposure and residents concerned about its risks. By serving as a tool for radiation risk communication, IREEs enabled stakeholders to promote both residents' safety and their sense of security.

Residents' sense of security is a particularly important consideration following a nuclear disaster. Honda et al. reported that providing a sense of security after the FDNPP accident contributed to reducing mental health risks among residents (21). Although concern about radiation exposure is a natural response, excessive anxiety can lead to psychological distress. Therefore, stakeholders must provide not only information regarding safety but also support that helps affected populations maintain wellbeing in their daily lives. Therefore, transparency regarding stakeholder activities related to residents' sense of security is essential.

Although stakeholder involvement was critical to promoting safety and security through IREEs, these efforts have not been adequately reported, and the associated activities remain unsystematized. Therefore, it is necessary to share the experiences of stakeholders involved in radiation risk communication related to IREEs and to ensure that this knowledge is passed on to future generations.

#### Perspective 3: documenting and sharing radiation risk communication practices related to the IREEs

Risk communication related to IREEs was typically conducted through face-to-face discussions based on WBC examination results (11). Consequently, although such communication was routinely performed, the interactions and the associated experiences were not systematically documented. Therefore, maintaining a fundamental record of these practices is important for preparedness for future nuclear disasters. To achieve this objective, accounts from individuals with actual experience, together with materials used during explanations, should be collected and organized to clarify the current state of radiation risk communication associated with IREEs. This information should be shared internationally under the theme “Safety and Security through IREEs in Fukushima.”

## Discussion

This report addresses not only the future of IREEs in Fukushima but also preparedness for IREEs in the event of nuclear disasters. The future of IREEs in Fukushima is directly linked to how long such examinations should continue following a nuclear disaster. Currently, no clear criteria exist for determining when resident IREEs can be discontinued. However, as mentioned earlier in the Introduction session, the IREEs are likely to be sustainable in the future only as a follow-up measure for individuals whose internal radiation dose from radioactive cesium exceeds 1 mSv. Confirmatory examinations may also be established for specific groups of individuals to verify the absence of related internal radiation exposure. For individuals who were previously included in the examination program but will no longer be covered, it is important to provide an appropriate explanation based on existing dose records, demonstrating that there is no contamination in the relevant area and no risk of internal exposure. For those who strongly wish to continue examinations, measurements may be taken even if their perception of the risk is incorrect.

Fifteen years after the FDNPP accident, these examinations have become increasingly distant in residents' memories. Whether IREEs remain necessary under such circumstances is an important question. This issue will be explored by examining future demand for IREEs and the role of radiation risk communication. In addition, preparedness for future nuclear disasters also requires consideration of both hardware and software aspects of IREE implementation. Practical expertise in operating WBC systems has been accumulated in Fukushima since the FDNPP accident. Organizing this knowledge will help ensure accurate IREE results and support the development of guidelines for incorporating protection against internal radiation exposure into future government disaster preparedness plans.

Many “Lessons learned from Fukushima” are embedded within the challenges and perspectives presented in this report. Co-authors who have been involved in Fukushima since immediately after the FDNPP accident are uniquely positioned to examine these experiences. Planned activities include: (i) evaluating IREE administration and conducting resident surveys to support Perspective 1 and improve accessibility so that residents from diverse backgrounds can undergo the IREEs with confidence; (ii) collecting operational knowledge from WBC operators at healthcare facilities to support Perspective 2, recognizing that accurate internal radiation exposure assessment is essential for radiation protection and risk reduction; (iii) documenting stakeholder activities related to radiation risk communication through analyses of practical experience and the literature. These activities will preserve and disseminate “Lessons learned from Fukushima” for future nuclear disaster preparedness.

**Figure 1:**
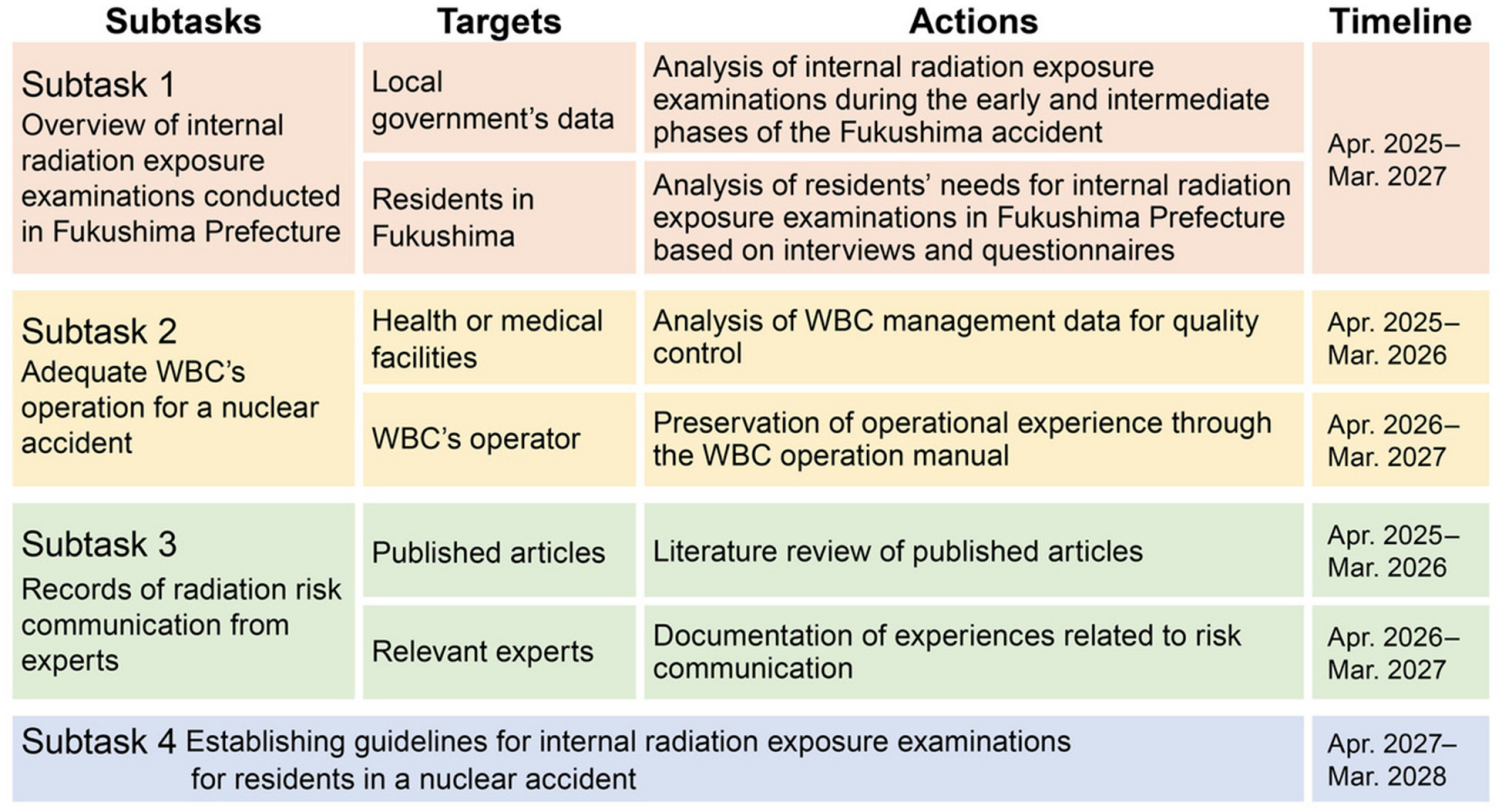
Objectives and corresponding actions with research periods for the four subtasks of the RECIPEx project.

A new initiative, the “Establishing Resident-Centered Implementation Policies for Internal Radiation Exposure Examinations” (RECIPEx) project, was launched to develop concrete responses based on these three perspectives. Running from April 2025 to March 2027, the RECIPEx project aims to establish a flexible and appropriate framework for IREEs in Fukushima while providing practical guidance for future nuclear accident responses. This project will also outline an appropriate strategy for making decisions regarding the IREE. This initiative aligns with the recommendation, “Adapt dosimetry and individual exposure monitoring to the situation,” from the Nuclear Emergency Situations-Improvement of Medical and Health Surveillance (SHAMISEN) project, which reported to the EU in 2021 on responses to residents during nuclear disasters (22, 23). The RECIPEx project comprises four subtasks, with subtasks 1–3 corresponding to the activities shown in Figure 1. This project's subtasks 1–3 responded to the Perspectives 1–3 in this report. The RECIPEx project was approved by the Ethics Review Committee of Fukushima Medical University (REC2024–211). Through comprehensive analyses of IREE-related activities and the collection of practical recommendations, the project seeks to preserve and transmit knowledge gained from the FDNPP accident under the title “Lessons learned from Fukushima.”

## Conclusion

This report aimed to identify key challenges associated with IREEs conducted for Fukushima residents following the FDNPP accident and to present perspectives for addressing these challenges. Three major challenges were identified, potential responses were discussed, and related activities within the RECIPEx project were outlined. Fifteen years after the FDNPP accident, opportunities to document the experiences and records of residents and stakeholders involved in IREEs are becoming increasingly limited. Efforts are therefore underway to compile analyses of IREE-related activities and collect practical knowledge in order to preserve and pass on the “Lessons learned from Fukushima” to future generations. The RECIPEx project has the potential to provide a framework that enables authorities to incorporate protection against internal radiation exposure into disaster prevention strategies, thereby strengthening public health and health crisis management.

## Data Availability

The original contributions presented in the study are included in the article/supplementary material, further inquiries can be directed to the corresponding author.
